# Supplementary material for: Obesity Is Indirectly Associated with Sudden Cardiac Arrest through Various Risk Factors
Source: J Clin Med. 2023 Mar 6;12(5):2068. doi: 10.3390/jcm12052068 (PMC10004688; doi:10.3390/jcm12052068)
Supplement: Supplementary file 1 [file jcm-12-02068-s001.zip › jcm-2204417-supplementary.pdf]

Obesity is Indirectly Associated with Sudden Cardiac Arrest through Various Risk Factors

Supplementary Table S1. Baseline characteristics of patients with and without SCA.

	SCA		
	**No**	**Yes**	**p-value**
	4,040,071	16,352	
**Male**	2,221,898 (55.0%)	11,633 (71.1%)	< 0.001
**Age (years)**	47.0 ± 14.1	62.0 ± 13.2	< 0.001
**Hypertension**	1,082,382 (26.8%)	9,331 (57.1%)	< 0.001
**Diabetes mellitus**	349,134 (8.6%)	4,264 (26.1%)	< 0.001
**Dyslipidemia**	732,983 (18.1%)	4,610 (28.2%)	< 0.001
**Chronic kidney disease**	275,854 (6.8%)	2,740 (16.8%)	< 0.001
**Body mass index (kg/m^2)**	23.7 ± 3.2	23.8 ± 3.4	0.138
**Waist circumference (cm)**	80.2 ± 9.5	83.5 ± 8.9	< 0.001
**Smoking**			< 0.001
Never-smoker	2,399,679 (59.4%)	7,916 (48.4%)	
Ex-smoker	581,485 (14.4%)	3,128 (19.1%)	
Current-smoker	1,058,907 (26.2%)	5,308 (32.5%)	
**Alcohol consumption**			< 0.001
Non-drinker	2,077,053 (51.4%)	9,534 (58.3%)	
Mild-drinker	1,641,427 (40.6%)	5,263 (32.2%)	
Heavy-drinker	321,591 (8.0%)	1,555 (9.5%)	
**Regular exercise**	733,609 (18.2%)	3,148 (19.3%)	< 0.001

**Income (lowest 20%)**	704,587 (17.4%)	3,075 (18.8%)	< 0.001
**Glucose (mg/dL)**	97.2 ± 23.8	110.0 ± 41.5	< 0.001
**Systolic blood pressure (mmHg)**	122.4 ± 15.0	129.3 ± 17.2	< 0.001
**Diastolic blood pressure (mmHg)**	76.3 ± 10.0	78.9 ± 11.0	< 0.001
**Cholesterol (mg/dL)**	195.3 ± 41.1	195.1 ± 44.3	0.549
**High-density lipoprotein (mg/dL)**	56.5 ± 32.9	53.6 ± 30.9	< 0.001
**Low-density lipoprotein (mg/dL)**	121.2 ± 214.2	115.0 ± 97.8	< 0.001
**eGFR (mL/min/1.73m^2)**	87.6 ± 44.9	80.4 ± 34.7	< 0.001

eGFR: estimated glomerular filtration rate; SCA: sudden cardiac arrest.

Supplementary Table S2. Impact of BMI and waist circumference on SCA according to metabolic syndrome

	n	SCA	Follow-up duration (person-years)	Incidence	Hazard ratio with 95% confidence interval					
					Univariate	Multivariate 1	Multivariate 2	Multivariate 3	Multivariate 4	Multivariate 5
**Participants who have all three diagnosis of hypertension, diabetes mellitus, and dyslipidemia**										
**BMI**										
BMI < 18.5	427	16	2,819	5.68	2.02 (1.22 – 3.34)	1.78 (1.08 – 2.95)	1.75 (1.06 – 2.90)	1.75 (1.06 – 2.90)	1.79 (1.08 – 2.97)	1.79 (1.08 – 2.95
18.5 ≤ BMI < 23	13.324	291	102,147	2.85	1 (reference)	1 (reference)	1 (reference)	1 (reference)	1 (reference)	1 (reference)
23 ≤ BMI < 25	18,455	282	146,315	1.93	0.68 (0.57 – 0.80)	0.70 (0.60 – 0.83)	0.71 (0.61 – 0.84)	0.71 (0.61 – 0.84)	0.71 (0.60 – 0.83)	0.70 (0.60 – 0.83)
25 ≤ BMI < 30	36,720	458	295,317	1.55	0.54 (0.47 – 0.63)	0.64 (0.55 – 0.74)	0.65 (0.56 – 0.75)	0.65 (0.56 – 0.75)	0.64 (0.55 – 0.74)	0.62 (0.53 – 0.72)
30 ≤ BMI	7,662	99	62,141	1.59	0.56 (0.44 – 0.70)	0.88 (0.69 – 1.10)	0.89 (0.71 – 1.12)	0.89 (0.71 – 1.12)	0.87 (0.69 – 1.10)	0.84 (0.66 – 1.06)
**Waist circumference (male / female; cm)**										
< 80 / 75	6,448	129	50,122	2.57	1.63 (1.32 – 2.02)	1.66 (1.35 – 2.06)	1.64 (1.32 – 2.03)	1.64 (1.32 – 2.03)	1.66 (1.34 – 2.06)	1.69 (1.37 – 2.09)
– 85 / 80	12,903	206	102,052	2.02	1.27 (1.06 – 1.53)	1.27 (1.06 – 1.53	1.27 (1.06 – 1.53)	1.27 (1.06 – 1.53)	1.28 (1.07 – 1.55)	1.30 (1.08 – 1.56)
– 90 / 85	19,395	246	155,024	1.59	1 (reference)	1 (reference)	1 (reference)	1 (reference)	1 (reference)	1 (reference)
– 95 / 90	17,877	254	142,385	1.78	1.13 (0.94 – 1.34)	1.12 (0.94 – 1.33)	1.11 (0.93 – 1.32)	1.11 (0.93 – 1.32)	1.11 (0.93 – 1.33)	1.10 (0.93 – 1.32)
– 100 / 95	11,308	164	90,481	1.81	1.14 (0.94 – 1.39)	1.19 (0.98 – 1.45)	1.19 (0.97 – 1.45)	1.19 (0.97 – 1.45)	1.17 (0.96 – 1.42)	1.15 (0.94 – 1.40)
≥ 100 / 95	8,657	147	68,674	2.14	1.35 (1.10 – 1.66)	1.49 (1.21 – 1.83)	1.47 (1.20 – 1.80)	1.47 (1.20 – 1.80)	1.44 (1.18 – 1.77)	1.41 (1.15 – 1.74)
**Counterpart (who have diagnosis none, one, or two of hypertension, diabetes mellitus, and dyslipidemia)**										
**BMI**										
BMI < 18.5	148,033	814	1,194,167	0.68	1.54 (1.43 – 1.66)	1.73 (1.61 – 1.87)	1.64 (1.52 – 1.76)	1.78 (1.65 – 1.92)	1.79 (1.66 – 1.93)	1.79 (1.66 – 1.93)
18.5 ≤ BMI < 23	1,566,329	5,725	12,864,605	0.45	1 (reference)	1 (reference)	1 (reference)	1 (reference)	1 (reference)	1 (reference)
23 ≤ BMI < 25	982,939	3,642	8,102,935	0.45	1.01 (0.97 – 1.05)	0.84 (0.81 – 0.87)	0.87 (0.83 – 0.90)	0.81 (0.77 – 0.84)	0.81 (0.77 – 0.84)	0.78 (0.75 – 0.82)
25 ≤ BMI < 30	1,145,678	4,457	9,447,808	0.47	1.06 (1.02 – 1.10)	0.88 (0.85 – 0.92)	0.92 (0.89 – 0.96)	0.81 (0.78 – 0.84)	0.80 (0.77 – 0.84)	0.75 (0.72 – 0.78)
30 ≤ BMI	136856	568	1,127,123	0.50	1.13 (1.04 – 1.23)	1.31 (1.20 – 1.43)	1.34 (1.23 – 1.46)	1.07 (0.98 – 1.17)	1.06 (0.97 – 1.16)	0.94 (0.87 – 1.03)
**Waist circumference (male / female; cm)**										
< 80 / 75	1,484,444	4,157	12,233,853	0.34	0.64 (0.61 – 0.67)	1.11 (1.07 – 1.17)	1.08 (1.03 – 1.13)	1.3 (1.24 – 1.36)	1.22 (1.16 – 1.27)	1.16 (1.11 – 1.22)
– 85 / 80	952,713	3,459	7,843,613	0.44	0.83 (0.79 – 0.87)	0.97 (0.93 –1.02)	0.96 (0.92 – 1.01)	1.03 (0.99 –1.08)	1.01 (0.96 – 1.06)	0.99 (0.94 – 1.04)
– 90 / 85	784,313	3,444	6,448,451	0.53	1 (reference)	1 (reference)	1 (reference)	1 (reference)	1 (reference)	1 (reference)
– 95 / 90	453,306	2,266	3,717,557	0.61	1.14 (1.08 – 1.20)	1.02 (0.97 – 1.08)	1.02 (0.96 – 1.08)	0.96 (0.91 – 1.01)	0.98 (0.93 – 1.03)	0.99 (0.94 – 1.05)

– 100 / 95	198,384	1,146	1,623,538	0.71	1.32 (1.24 – 1.41)	1.15 (1.08 – 1.23)	1.15 (1.08 – 1.23)	1.02 (0.95 – 1.09)	1.06 (0.99 – 1.13)	1.09 (1.02 – 1.16)
≥ 100 / 95	106,675	734	869,627	0.84	1.58 (1.46 – 1.71)	1.48 (1.36 – 1.60)	1.46 (1.35 – 1.58)	1.2 (1.11 – 1.30)	1.27 (1.17 – 1.38)	1.31 (1.21 – 1.42)

Incidence is per 1,000 person-years follow-up.

BMI: body-mass-index; SCA: sudden cardiac arrest; ɣ-GTP: gamma-glutamyltransferase.

Multivariate model 1: adjusted for age and sex.

Multivariate model 2: adjusted for model 1 plus smoking, alcohol, regular exercise, and income.

Multivariate model 3: adjusted for model 2 plus hypertension, diabetes mellitus, dyslipidemia.

Multivariate model 4: adjusted for model 2 plus hypertension, diabetes mellitus, dyslipidemia, and chronic kidney disease.

Multivariate model 5: adjusted for model 4 plus ɣ-GTP.
